# Neural determinants of human goal-directed vs. habitual action control and their relation to trait motivation

**DOI:** 10.1038/s41598-017-06284-y

**Published:** 2017-07-20

**Authors:** Hamdi Eryilmaz, Anais Rodriguez-Thompson, Alexandra S. Tanner, Madeline Giegold, Franklin C. Huntington, Joshua L. Roffman

**Affiliations:** Department of Psychiatry, Massachusetts General Hospital and Harvard Medical School, Boston, MA USA

## Abstract

Instrumental learning is mediated by goal-directed and habit systems in the brain. While rodent studies implicate distinct prefrontal/striatal regions in goal-directed and habit learning, neural systems underpinning these two processes in humans remain poorly understood. Here, using a validated discrimination learning task that distinguishes goal-directed learning from habit learning in 72 subjects in fMRI, we investigated the corticostriatal correlates of goal-directed learning and tested whether brain activation during learning is associated with trait motivation and behavioral performance in the post-learning test phase. Participants showed enhanced activation in medial prefrontal and posterior cingulate cortices during goal-directed action selection in the training phase, whereas habitual action selection activated bilateral insula, bilateral dorsal caudate and left precentral gyrus. In addition, early phase of learning was associated with increased activation in the frontoparietal control network and dorsal striatum, whereas default mode regions depicted increased activation in the late phase. Finally, avoidance motivation scores measured by Behavioral Inhibition/Activation System (BIS/BAS) correlated with accuracy during goal-directed learning and showed a nominally significant correlation with activation in dorsomedial prefrontal cortex during goal-directed acquisition of stimuli. These findings reveal the temporal dynamics of instrumental behavior and suggest that avoidance motivation predicts performance and brain activity during goal-directed learning.

## Introduction

Instrumental behavior is supported by goal-directed and habit systems in the brain^1, 2^. The goal-directed system encodes the relationship between action and outcome in order to select actions that are in accordance with the current desires of the organism^3, 4^. This system is advantageous when exploring different options and assessing the subjective value of a particular outcome are relevant for subsequent action selection. When the relationship between representations of actions and outcomes is established, the habit system, which encodes stimulus-response associations, weighs in to guide action selection^5, 6^. The habit system increases efficiency at the expense of flexibility, whereas the goal-directed system optimizes action selection in a way sensitive to the current value of the outcome. Based on this account, there is a balance between these two systems, which serves to optimize action selection, increase efficiency and maintain flexibility. Importantly, disruption in such balance has been linked to instrumental learning deficits in neuropsychiatric diseases such as obsessive-compulsive disorder, where patients rely disproportionately on habitual action control^7^.

Neural systems underpinning the dynamics of this balance in humans remain largely elusive despite substantial evidence from rodent studies indicating that distinct striatal systems mediate goal-directed and habitual learning in coordination with the prefrontal cortex^8–10^. The substantial input that the striatum receives from the cortex and other subcortical structures^11^, allows this region to access and integrate contextual, motivational and motor information to select actions based on the organism’s current desires. Indeed, studies in rodents provided evidence for a dorsomedial striatal/orbitofrontal system mediating goal-directed learning^8, 12^, and a dorsolateral striatal system implicated in habit learning^13^. Determining the homologues of these systems in humans has been more challenging since the behavioral tests that determine whether performed actions are goal-directed and habitual have not been as efficient in humans. Nevertheless, several human fMRI studies using various reinforcement learning tasks have implicated ventral medial prefrontal cortex (vmPFC) in encoding the value of a predicted reward linked to a specific action^14, 15^, whereas posterior putamen and caudate have been demonstrated to be involved in habitual action control^16, 17^. Further, the idea that the activity is shifted from ventral to dorsal striatum as actions become more habitual has been prominent mostly based on the evidence from rodent studies^5^, however, it is unclear whether this is the case in humans.

A robust paradigm was demonstrated to distinguish goal-directed and habit learning in humans by introducing incongruity during acquisition and creating a baseline for habit learning^3, 18^. In the present study, we leveraged this task in fMRI to identify the brain regions that are associated with acquisition of goal-directed and habitual behavior. In addition, using previously validated behavioral post-training tests^7^, we examined individual differences in goal-directed vs. habitual action control once the appropriate actions were acquired. Finally, we investigated whether any differential brain activity between goal-directed and habit learning is associated with individual approach/avoidance motivation traits using Behavioral Inhibition/Activation System (BIS/BAS) scales^19^. Both systems are thought to influence the motivational state of an individual and consequent action selection^20, 21^.

## Methods

### Participants

96 healthy adults with no history of neurological or psychiatric disorders participated in the present study. 12 participants were excluded from the analysis for either not performing above chance during the learning phase in the scanner or misunderstanding the post-scan tests, 7 because of technical issues during the experiment, 3 due to artifacts in the imaging data and 2 due to average head motion greater than 0.1 mm per TR, leaving 72 participants (35 males, age 18–35 years, mean 24.7 years) for the analysis. All participants provided written informed consent. The experimental protocol was approved by Partners Healthcare Human Research Committee and all procedures were carried out in accordance with the guidelines.

### Experimental Procedure

As part of a larger study, participants underwent an interview for psychiatric screening, completed North American Adult Reading Test (NAART) and BIS/BAS questionnaire prior to the fMRI scan. They received instructions for a discrimination learning task to be performed in the scanner. In the scanner, participants performed a single run of the discrimination learning task (14 min). After the learning task in the scanner, they completed two tasks outside the scanner, which tested action selection solely based on outcomes (Outcome Devaluation Test) and goal-directed vs. habitual responding to the stimuli from the learning phase (Slip Test). The present study reports behavioral and imaging data obtained during the discrimination learning task, the two post-scan tests, demographics and questionnaire data on BIS/BAS.

### Discrimination Learning Task

**Figure 1:**
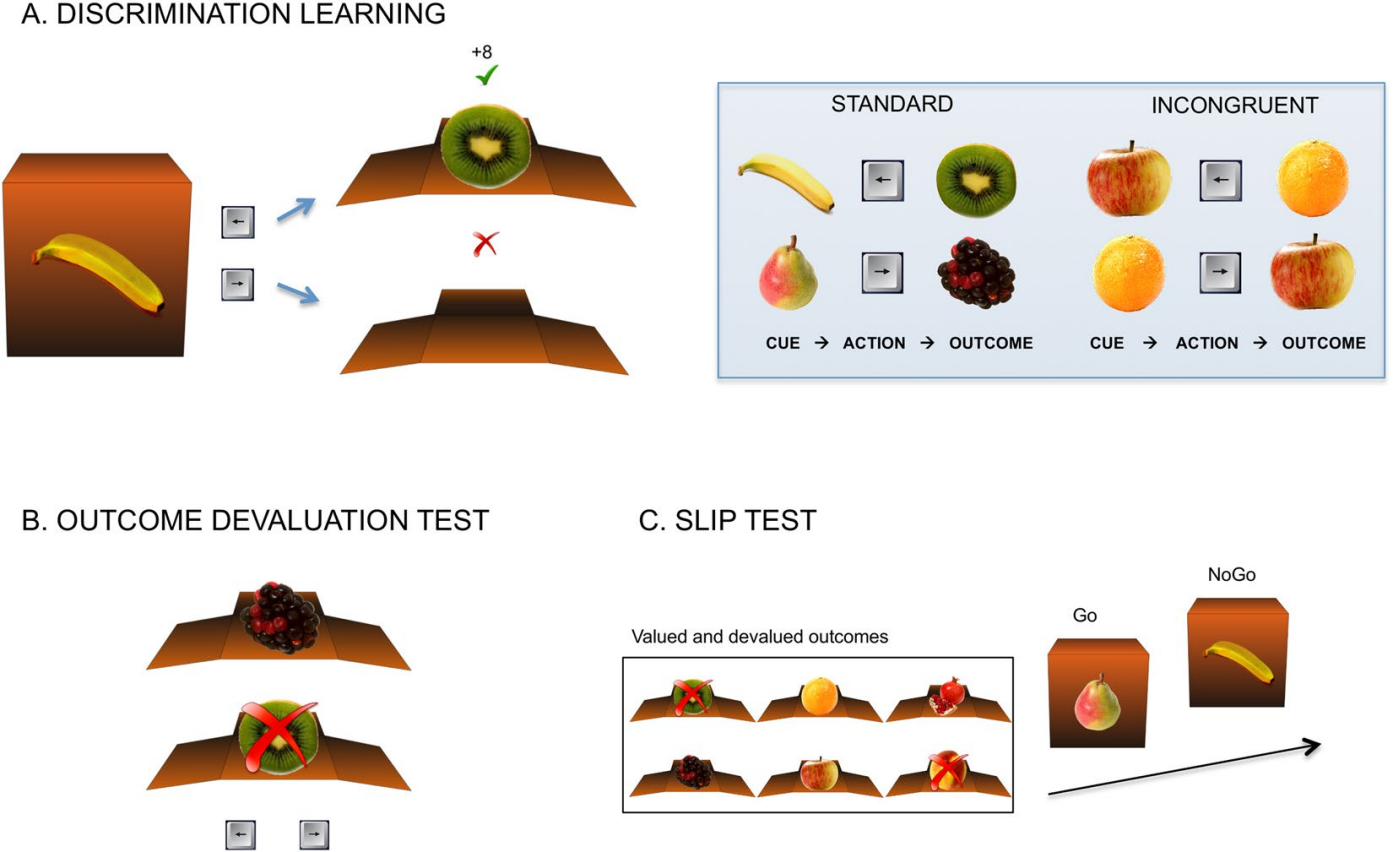
Experimental paradigm. (**A**) The Discrimination Learning Task performed in the scanner is shown. In the illustrated example, subjects are presented with a closed box and a banana picture (cue) in front of it. They are asked to press the key associated with the banana, which they learn via feedback presented after they make their choice. If the subject presses the correct key, then the box opens with another fruit inside and a number showing the points earned. If the wrong key is pressed or response is omitted, an empty box opens. In the standard condition, a given fruit is only used either as cue or as outcome (but not as both), whereas in the incongruent condition, a given fruit is used as cue in some trials and as outcome in others. The task also includes a control condition, in which an arrow is presented above the box instead of a fruit and shows the direction of the key to be pressed. The box then opens with a blue circle displayed above it. (**B**) The Outcome Devaluation Test is shown. In this example trial, two open boxes are presented. The “X” on kiwi indicates that this fruit is devalued and no longer offers points. Therefore, the correct response is the right key, which led participants to the valuable outcome (blackberry) during the learning phase. (**C**) The Slip Test is illustrated. At the beginning of each block, subjects are presented with outcome fruits to obtain in the upcoming set of trials. Unlike the learning task, 2 fruits (out of 6 in a block) are devalued and collecting them would result in subtraction of points. Those devalued outcomes are marked with ‘X’s. In the example shown, kiwi is an outcome to avoid, therefore, when presented with a banana, subjects should not press any key to avoid loss of points. The subjects are asked to collect the valuable fruits. Since the pear leads to a valuable fruit (blackberry), the subjects should press the correct key (right) in response to pear. The fruit images in this figure were obtained from the *food-pics database* ^38^ for illustration purpose and their brightness was adapted for this figure. The license agreement is available on https://creativecommons.org/licenses/by/3.0/legalcode.

We used a variant of a previously developed discrimination learning task^3^. In this task, participants learn the association between pairs of fruits and a key press to earn monetary reward (Fig. 1A). During instructions, subjects were informed that they would be remunerated proportional to their total points earned in all three tasks. In each trial, a closed box was shown with a fruit picture on it (2 s). This cue fruit signaled another fruit inside the box. In order to obtain the fruit inside the box and earn points, participants had to press the correct key (left or right key on a button box) within 2 s. They were asked to respond as fast as possible and informed that key presses made after the cue disappears would not be valid. The box then opened showing the outcome fruit (1 s) in the box with the points earned (if the correct key was pressed) or the empty box (if the response was incorrect). Reward amount was pseudorandomized between 3–9 points per trial. Each pair of fruits was assigned a specific key, which participants learned by trial and error. The task involved 3 conditions: standard, incongruent, and control. In the *standard* condition, a given fruit could be either a cue or an outcome (but not both), whereas in the *incongruent* condition, a given fruit appeared both as cues and as outcomes in different trials. This incongruency was previously shown to make goal-directed responding disadvantageous^18^. In essence, the incongruent condition creates a conflict between the action triggered by an event acting as a cue and the action associated with the same event when it acts as an outcome. For example, in the apple-orange pair shown in Fig. 1A, the apple is associated with the left key response when it serves as a cue, whereas it is associated with the right key response when it serves as an outcome. Therefore, concurrently forming action-outcome and stimulus-response associations would lead to a conflict between left and right key responses. This concept was previously validated with an outcome devaluation procedure^3^. It was shown that in an outcome-cued test, responding to stimuli from the incongruent condition (unlike the standard condition) was unaffected by outcome devaluation, which showed that learning was mediated by stimulus-response associations. Since participants rely on habitual responding during incongruent trials to successfully perform the task, this condition provides an effective baseline for habit learning. Finally, a *control* condition consisting of the visual and motor aspects of the task was also included. In this condition, an arrow was presented on top of the box instead of a fruit picture, which signaled the direction of the arrow key to be pressed for that trial. The key press then opened the box and a blue circle was presented with an empty box. No points were earned in the control trials. Intertrial interval (ITI) was pseudorandomized between 2.85–4.75 s. Standard, incongruent and control trials were separately presented in blocks, each of which consisted of 8 trials. Four pairs of fruits were used in each of the standard and incongruent conditions and they were presented in randomized order within a block. Each task block was followed by a 12 s fixation block. Participants went through 6 standard, 6 incongruent, and 3 control blocks.

### Outcome Devaluation Test

In this post-scan test, participants were presented with only the outcomes (without cues) from the learning phase and asked to press the key that led them to those outcomes during learning. Therefore, this task allowed us to test outcome-based responding. In each trial, two vertically positioned open boxes (with fruits in them) were presented to the participants (Fig. 1B). They were informed that one of these fruits was earned by pressing the left key, and the other fruit was earned by pressing the right key during the learning phase. Although both fruits earned them points during the learning phase, one of these fruits was now devalued (worth no points), which was indicated by an “X” in red. Participants were instructed to press the key, which previously led them to the currently valuable fruit. No feedback was provided during this test. Participants responded to 16 trials (8 from standard, 8 from incongruent pairs) in a randomized order.

### Slip Test

In this second post-scan test, participants performed a task similar to the discrimination learning task they performed in the scanner (Fig. 1C). However, in the current task they were asked to avoid obtaining certain fruits by omitting response. This task has proven to be robust in testing the ability to flexibly adapt action selection^7^. At the beginning of each block, participants were presented with six outcome fruits from the training stage (8 s). Four of these fruits were marked as valuable (to be collected) and two were marked as devalued (not to be collected) for that block. Next, a series of closed boxes (with fruits in front of them) was presented (1 s). Participants were asked to press the correct key (they learned during the training phase) associated with the cue if the expected outcome fruit is a valuable one, whereas they were asked to withhold their response if the expected outcome was devalued. If they pressed the correct key when the outcome was valuable, they earned 2 points, however, if they pressed any key when the outcome was devalued they lost 1 point. Therefore, in order to avoid losing points they needed to refrain from responding when presented with a fruit for which the outcome was devalued. In essence, goal-directed and habit systems compete for action control in this test. As the participants developed stimulus-response associations during the training phase, withholding response in this task required goal-directed action control. If a participant fails to employ goal-directed action control, he/she would be more likely to commit a slip at cues with devalued outcomes. Participants completed 3 standard and 3 incongruent blocks with each block consisting of 16 trials.

### MRI acquisition

Functional and structural MRI data were collected in a Siemens 3T Skyra system. EPI images were acquired in a single run while the participants performed the Discrimination Learning Task with the following parameters: repetition time/echo time/flip angle = 1900 ms/30 ms/90°, in-plane resolution = 3.6 × 3.6 mm, slice thickness = 3 mm. A high-resolution T1 image (repetition time/echo time/flip angle = 2200 ms/1.54 ms/7°) with 144 axial slices and an isotropic voxel size of 1.2 × 1.2 × 1.2 mm was also acquired.

### fMRI preprocessing

We used Freesurfer v5.3 for all steps of fMRI preprocessing (https://surfer.nmr.mgh.harvard.edu/). Functional images were realigned to the middle time point image, normalized to the MNI305 template (2 × 2 × 2 mm) using a boundary-based registration model^22^. All functional images were then smoothed using a 6 mm Gaussian kernel.

### Main GLM design

Preprocessed fMRI data were analyzed in Freesurfer Functional Analysis Stream (FS-FAST). We analyzed activations in response to both cue and outcome presentation for *standard*, *incongruent*, and *control* conditions. Therefore, in our GLM design, each of Standard Cue, Incongruent Cue, Standard Outcome, Incongruent Outcome, Control Cue, and Control Outcome trials were included as main regressors. In addition, a parametric modulator (for each of the conditions Standard Outcome and Incongruent Outcome) reflecting the reward amount in each trial was added. These regressors helped account for activations that arose exclusively in response to the reward amount. Epochs were convolved with a gamma hemodynamic response function (delay = 2.25 s, dispersion = 1.25 s)^23^. Each cue or outcome was modeled as an epoch (2 s for cues and 1 s for outcomes) in the design matrix. Finally, we also added six motion parameters to account for movement artifacts. All regressors were then submitted to a standard GLM analysis in Freesurfer.

The contrasts ‘Standard Cue vs. Incongruent Cue’ and ‘Incongruent Cue vs. Standard Cue’ examined activation arising in response to goal-directed and habitual action selection respectively. Similarly, ‘Standard Outcome vs. Incongruent Outcome’ and ‘Incongruent Outcome vs. Standard Outcome’ revealed selective brain responses to rewarding outcomes following goal-directed and habitual action selection respectively. Once parameter estimates were computed for each subject, they were entered into a second-level random-effects group analysis. For all GLM analyses, we used 10,000 Monte Carlo simulations to correct for multiple comparisons in the whole brain. Prior to simulations, the maps were thresholded at p < 0.001. Monte Carlo simulations then determined the likelihood that resulting clusters would be found by chance. The corrected p-value was set to 0.05 (two-tailed).

### Early vs. Late Phase of Learning

In order to compare brain activation during early and late phases of learning, we implemented an additional categorical GLM design, in which we included first 2 blocks, middle 2 blocks and last 2 blocks of the Standard and Incongruent conditions during learning (both cue and outcomes) as separate regressors. Control Cue and Control Outcome were also added as main regressors. As in the main GLM design, six motion parameters were also included as nuisance regressors. The contrasts ‘Early Standard Cue vs. Late Standard Cue’ and ‘Early Incongruent Cue vs. Late Incongruent Cue’ determined activations arising in response to action selection during early phases of learning (vs. late phase) for each of the main conditions. Similar contrasts were used to investigate activation during early and late outcomes (i.e, Early Standard Outcome vs. Late Standard Outcome).

### Behavioral correlations

Using a ROI approach, we performed a correlation analysis to assess whether the strength of discrimination-selective brain activation during the learning phase in preselected ROIs (based on GLM results) is associated with behavioral measures from the test phase (i.e., goal directedness). In addition, we computed the correlation between learning-related brain activation and BAS-Drive or BIS scores. For these analyses, we used False Discovery Rate (FDR) correction to control for multiple comparisons among 8 ROIs (q = 0.05). Finally, we also computed the correlation between BAS-Drive/BIS scores and accuracy/reaction time measures to test for an influence of approach/avoidance motivation on task performance.

### Gender analysis

We conducted an exploratory analysis to test for potential gender differences in discrimination-specific brain activation, demographic variables age and education level, and behavioral variables such as learning accuracy, BAS-Drive, BIS, and goal-directedness in the Slip Test. A two-sample t-test determined significance of difference between 35 males and 37 females.

### Data availability

The datasets generated during and/or analyzed during the current study are available from the corresponding author on reasonable request.

## Results

### Behavioral results

**Figure 2:**
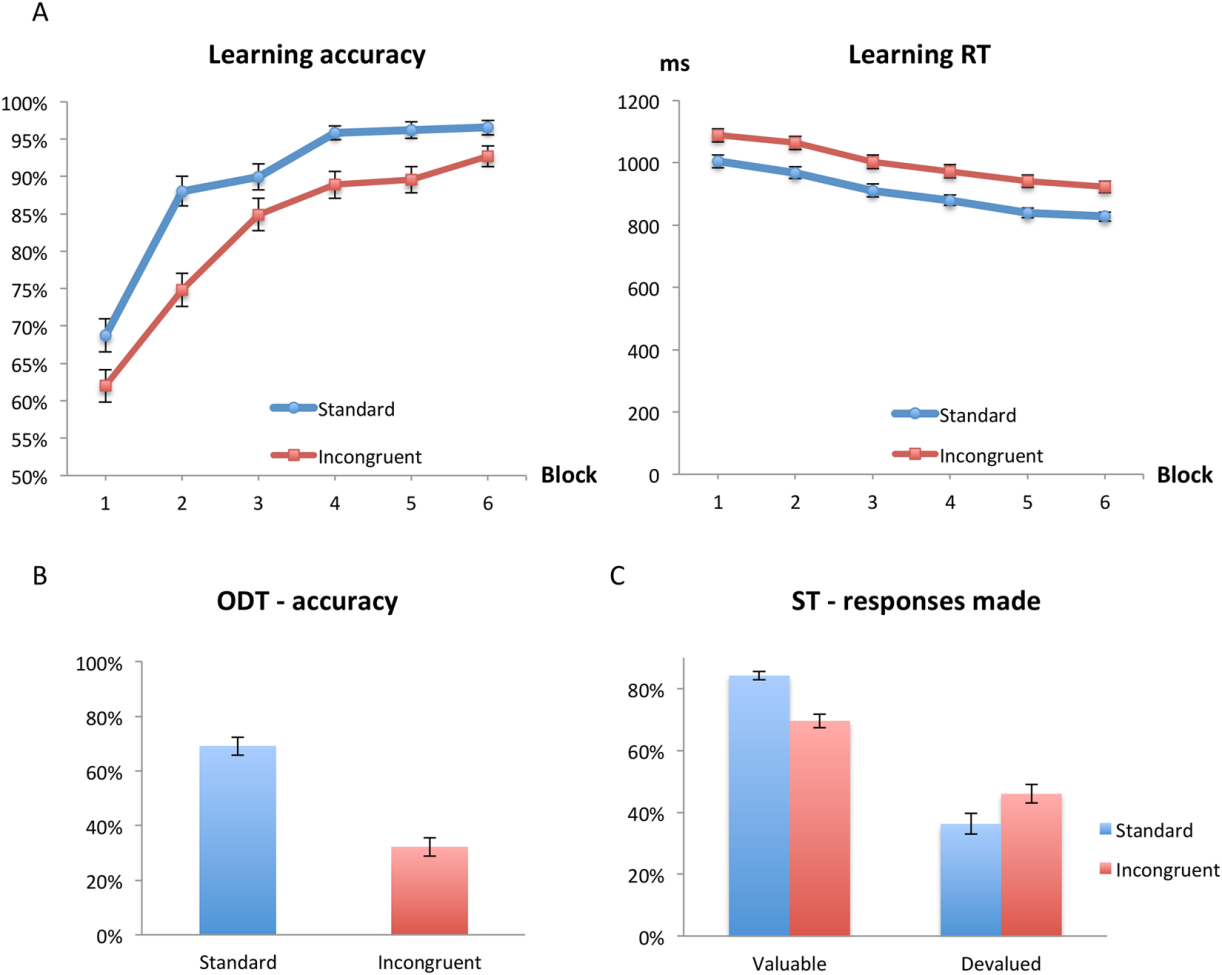
Behavioral results. (**A**) Subjects’ accuracy and reaction time are displayed for the learning phase. Subjects rapidly learned both discriminations and reached high levels of accuracy at the end of six blocks. Their responses gradually became faster in both conditions, and overall they were significantly faster in the standard condition. (**B**) In the Outcome Devaluation Test, subjects’ responses to outcomes from standard pairs were more accurate (than incongruent). (**C**) Percentage of the responses made during the Slip Test is shown for valuable/devalued outcomes in both conditions. Subjects responded less often when the outcomes were devalued in both conditions, however, there was a significant interaction of Value X Discrimination, i.e., subjects responded for devalued outcomes more often and responded for valuable outcomes less often during incongruent trials. Error bars represent the standard error of the mean. Abbreviations: RT: reaction time, ODT: Outcome Devaluation Test, ST: Slip Test.

At the end of six learning blocks (in each of the conditions *standard* and *incongruent*) during the discrimination learning task in the scanner, participants demonstrated high levels of accuracy (Fig. 2A), which confirms that they successfully learned the association between the fruit pairs and the correct key press in both conditions. We performed an ANOVA with the within-subject factors Block (1, 2, 3, 4, 5, 6) X Discrimination (standard, incongruent). This analysis revealed a significant interaction of block and discrimination (F(5, 355) = 2.77, p < 0.05). Post hoc analyses confirmed a highly significant effect of both block (F(5, 355) = 105.79, p < 0.001) and discrimination (F(1, 71) = 54.01, p < 0.001), with lower overall accuracy in the incongruent condition than in the standard condition. Participants’ responses gradually became faster in both conditions. However, they responded consistently more slowly during incongruent pairs. An ANOVA with the within-subject factors Block X Discrimination revealed a highly significant effect of block (F(5, 355) = 44.25, p < 0.001) and discrimination (F(1, 71) = 111.92, p < 0.001) on reaction time, but no interaction.

In the Outcome Devaluation Test (performed after the scan), participants responded solely based on the outcomes and without the cues from the learning phase in the scanner. This analysis revealed a significant effect of discrimination confirmed by a paired t-test (p < 0.001). Accuracy was higher for the standard condition (Fig. 2B). In addition, we found a significant effect of reaction time (p < 0.001), where responses to outcomes from the standard pairs were faster than to those from the incongruent ones.

Slip Test measured participants’ ability to flexibly adapt their responding when certain outcomes became devalued and undesired since they resulted in loss of points. We observed a significant effect of devaluation in both types of discrimination (p < 0.001 for both standard and incongruent trials) as well as an interaction of Value X Discrimination (F(1, 71) = 39.57, p < 0.001), which was driven by greater number of responses for devalued outcomes during incongruent trials. In an additional analysis, we computed, for each participant, ‘goal-directedness’ as [responses made for valuable outcomes – responses made for devalued outcomes]. Therefore, higher scores reflect goal-directed responding, whereas lower scores indicate reliance on habitual responding. A paired t-test revealed a significant effect of discrimination (p < 0.001), indicating that participants were better able to adapt their responses during standard condition compared to the incongruent one (Fig. 2C). In other words, they were more vulnerable to slips when responding to incongruent pairs suggesting the influence of habitual action control. Finally, another test also revealed a significant effect of discrimination on reaction time (p < 0.001), where subjects responded faster during standard trials than during incongruent ones.

### GLM results

Here we report the differences in activation between the standard and incongruent conditions during cue and outcome presentation. For the results of analyses showing general effects of learning (standard vs. control, incongruent vs. control), see Fig. S1 and Supplementary Results.

#### Cue presentation

**Figure 3:**
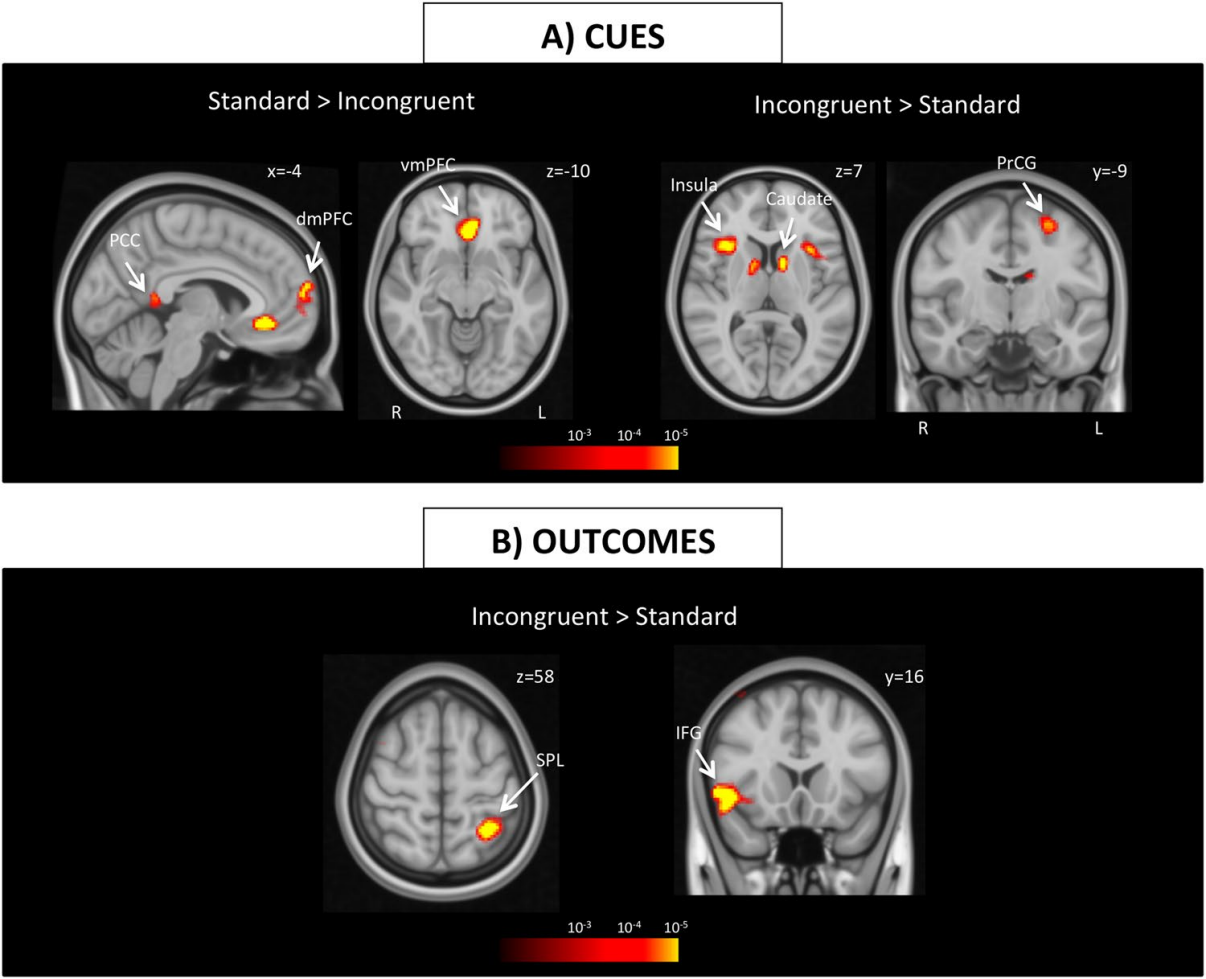
Brain activation during cue and outcome presentation. (**A**) Brain regions that were more active during standard and incongruent cues are depicted. PCC and medial prefrontal areas demonstrated greater activation during standard cues, whereas bilateral insula, dorsal caudate and left precentral gyrus showed enhanced activation during incongruent cues. (**B**) No region reached significance for Standard > Incongruent outcomes, however, left SPL and right inferior frontal gyrus demonstrated stronger activation during incongruent outcomes. All clusters displayed on the maps survive correction for multiple comparisons using a cluster-defining threshold of p < 0.001 and 10,000 Monte Carlo simulations at clusterwise p < 0.05. Abbreviations: dmPFC: dorsomedial prefrontal cortex, IFG: inferior frontal gyrus, PCC: posterior cingulate cortex, PrCG: precentral gyrus, SPL: superior parietal lobule, vmPFC: ventral medial prefrontal cortex.

We first identified regions responding differentially to standard and incongruent cues in the learning phase. During cue events, subjects viewed a fruit in front of the box, remembered the associated key with that fruit and executed a motor response. Subjects demonstrated enhanced activation in vmPFC, dorsomedial prefrontal cortex (dmPFC), and posterior cingulate cortex (PCC) during standard cues (Fig. 3A and Table 1). Conversely, bilateral insula, bilateral dorsal caudate, and left precentral gyrus showed elevated activation in response to incongruent cues.

**Table 1:** List of brain regions showing significant differential activation in response to standard vs. incongruent cues and outcomes.

Region (ROI)	Side	Coordinates			p-value	Cluster size (N)
		x	y	z		
**Standard > Incongruent CUE**						
vmPFC	L	−4	33	−9	4.4 × 10^−8^	482
dmPFC	L	−2	63	17	4.4 × 10^−6^	320
PCC	L	−8	−47	5	9.6 × 10^−6^	403
**Incongruent > Standard CUE**						
Insula	L	−34	15	3	2.2 × 10^−6^	422
Insula	R	34	19	5	2 × 10^−6^	616
Caudate	L	−10	3	3	1.2 × 10^−6^	274
Caudate	R	12	7	4	1.5 × 10^−5^	343
PrCG	L	−26	−9	59	2.2 × 10^−5^	393
**Incongruent > Standard OUTCOME**						
IFG	R	48	19	−5	7.8 × 10^−8^	771
Cuneus	L	−8	−91	−1	2.3 × 10^−7^	1701
Cuneus	R	6	−70	13	4.6 × 10^−6^	1626
SPL	L	−32	−51	61	2.6 × 10^−7^	379

Cluster size indicates the number of voxels surviving Monte Carlo corrected threshold. Coordinates and the p value are shown for the peak voxel in each region. Abbreviations: dmPFC: dorsomedial prefrontal cortex, IFG: inferior frontal gyrus, PCC: posterior cingulate cortex, PrCG: precentral gyrus, SPL: superior parietal lobule, vmPFC: ventral medial prefrontal cortex.

#### Outcome presentation

We then determined regions with differential responses to standard and incongruent outcomes. During outcomes, subjects viewed the outcome fruit and the points earned for that trial. No region reached significance at ‘Standard Outcome vs. Incongruent Outcome’. In contrast, right inferior frontal gyrus, bilateral cuneus, and left superior parietal lobule (SPL) were more strongly activated during incongruent outcomes compared to standard ones (Fig. 3B and Table 1).

#### Early vs. late phase

**Figure 4:**
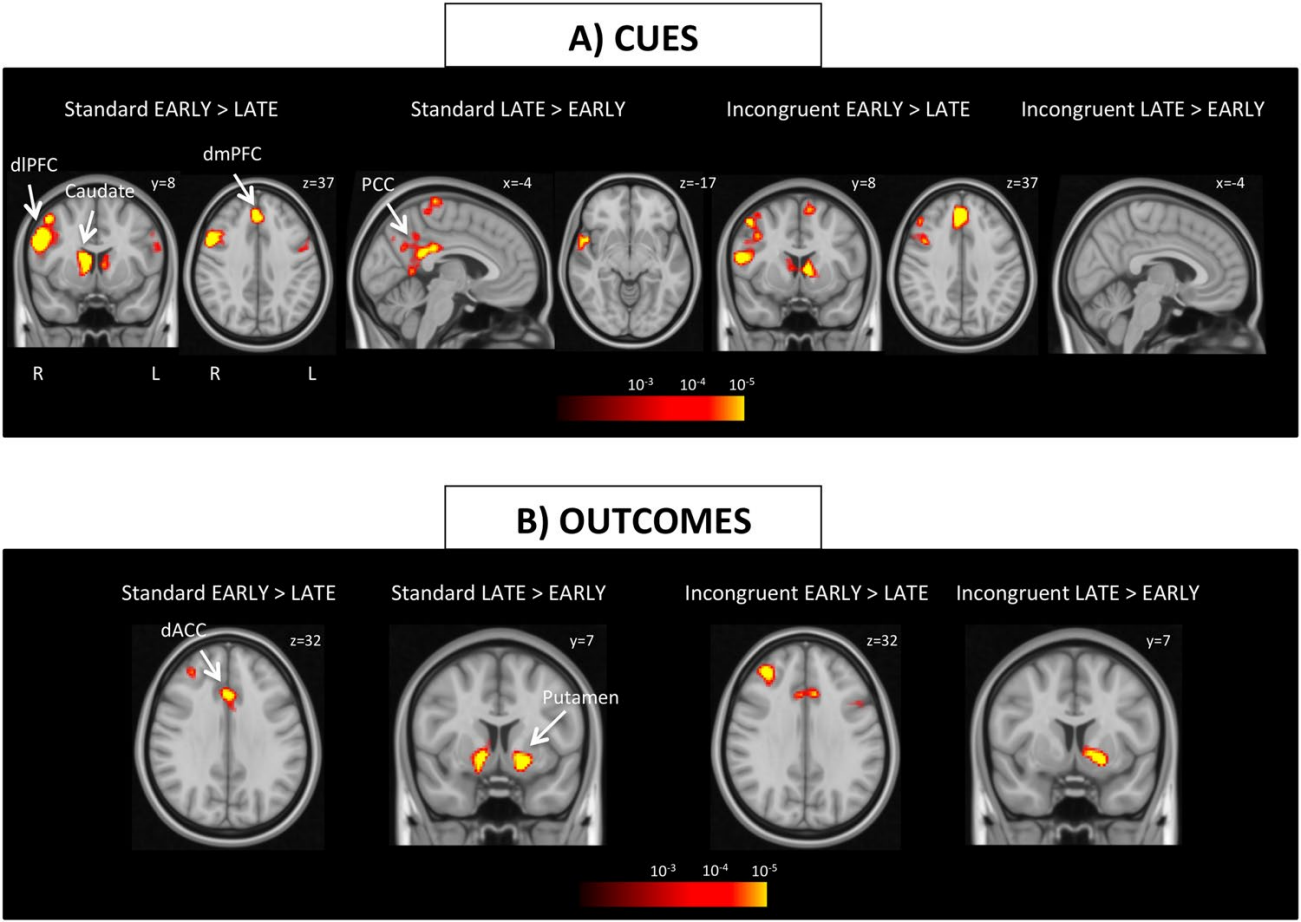
Brain activation during early and late learning phases. (**A**) Regions depicting stronger activation in early (first 2 blocks) versus late phases (last 2 blocks) of learning. Stronger activation was observed in bilateral dorsal caudate, bilateral insula, dmPFC, and right dlPFC during cue presentation in the early phase in both standard and incongruent trials. Conversely, in the late phase, PCC, bilateral postcentral gyrus, and right superior temporal gyrus showed enhanced activation only during standard trials. During outcome presentation, stronger activation was observed in dACC and right dlPFC in the early phase, whereas ventral striatum activation was enhanced in the late phase. All clusters displayed on the maps survive correction for multiple comparisons using a cluster-defining threshold of p < 0.001 and 10,000 Monte Carlo simulations at clusterwise p < 0.05. Abbreviations: dACC: dorsal anterior cingulate cortex, dlPFC: dorsolateral prefrontal cortex, dmPFC: dorsomedial prefrontal cortex, PCC: posterior cingulate cortex.

In order to assess the temporal dynamics of learning, we performed an additional categorical analysis, in which we compared brain activation during the first and the last two blocks for both standard and incongruent conditions. Subjects demonstrated stronger activation in bilateral dorsal caudate, bilateral insula, dmPFC, and right dlPFC during cue presentation in the early phase of learning relative to the late phase in each of the main conditions. Conversely, PCC, bilateral postcentral gyrus, and right superior temporal gyrus showed enhanced activation during late phase of learning relative to the early phase during standard trials only (Fig. 4A and Table S1). During outcome presentation in standard trials, dorsal anterior cingulate cortex (dACC) and right dlPFC demonstrated stronger activation in early phases, whereas bilateral ventral striatum activation was dominant in late phases (possibly reflecting higher points won). Incongruent condition depicted similar results (Fig. 4B and Table S2).

### Behavioral correlations

To assess whether trait motivation and behavioral performance (goal-directedness) during the test phase predicted differential activation during goal-directed and habit learning, individual scores from BIS and BAS-Drive scales and goal-directedness scores from the Slip Test were correlated with percent fMRI signal change at main effect of discrimination (‘Standard Cue vs. Incongruent Cue’ or ‘Standard Outcome vs. Incongruent Outcome’) extracted from 8 ROIs (regions resulting from the main cue contrasts in Table 1). None of the correlations between the behavioral measures and brain activation survived FDR correction. BIS scores showed nominally significant positive correlation with activation in dmPFC at Standard vs. Incongruent Cue (p = 0.025, r = 0.26), and negative correlation with activation in right caudate at Standard vs. Incongruent Outcome (p = 0.041, r = −0.24). The degree of goal-directed responding during the Slip Test showed nominally significant negative correlation with activation in dmPFC at Standard vs. Incongruent Outcome (p = 0.042, r = −0.24). Finally, BIS scores were significantly correlated with accuracy during acquisition of standard pairs (but not incongruent ones) in the learning phase (p = 0.016, r = 0.28). See Fig. S2 for scatterplots.

### Gender effects

We also performed an exploratory analysis to test for potential gender differences in differential activation between goal-directed and habit learning and in behavioral measures such as learning accuracy, BAS-Drive, BIS, and goal-directedness in the Slip Test. The comparison of 35 males and 37 females resulted in no significant discrimination-specific activation (standard vs. incongruent) during neither cues nor outcomes. In addition, age and highest completed education levels did not differ between the two gender groups. However, females showed relatively higher goal-directedness during the Slip Test (p < 0.003). There was no significant gender effect in learning accuracy, BAS-Drive and BIS scores.

## Discussion

Building upon previous animal and human research that identified two distinct systems mediating instrumental learning and using a validated task in fMRI, we investigated the cortical and subcortical brain areas that are involved in goal-directed and habit learning. Our findings reveal the central role of medial prefrontal cortex in goal-directed learning and implicate insula and dorsal striatum in processes that mimic habit learning. The analysis contrasting early and late phases of learning further demonstrates that as actions become automatic, the striatum and cortical dorsal attention network are less involved in action selection. Finally, the present results also suggest that avoidance motivation promotes goal-directed (as opposed to habitual) learning.

Substantial evidence from rodent and human literature suggests that reward-motivated behavior is mediated by the interplay between a system that controls the acquisition of goal-directed actions, and another system controlling the acquisition of habits^1^. The former builds associations between actions and the outcomes they lead to^2^, whereas in the latter associations are established between stimuli and responses^5^. Therefore, both systems have distinct ways of selecting actions, which is typically formalized via a process called outcome devaluation. Based on this account, the goal-directed system selects actions in a way that is sensitive to the changes in the value of the outcome, whereas the habit system remains insensitive to such changes. Our post-scan tests provided direct evidence with regard to this distinction. We first used the Outcome Devaluation Test, in which subjects had to select between two actions only based on the current value of the outcomes (without help from the cues). In line with previous research^18^, this test revealed that subjects were more accurate when responding to the outcomes from the standard (compared to incongruent) condition of the learning task, suggesting better action-outcome encoding for this set of stimuli. Furthermore, the Slip Test demonstrated that when the outcome values changed, subjects committed more slips when responding to cues from incongruent pairs (compared to standard). This finding is also consistent with previous work^7^ and underlines insensitivity to changes in outcome value in incongruent pairs. Considering the very high accuracy rates for both conditions at the end of the learning phase (therefore ruling out between-condition performance differences), these data support the idea of dual learning mechanisms and influence of action-outcome associations in standard pairs and that of stimulus-response associations in incongruent pairs.

Our imaging results identified a central role for medial prefrontal cortex in goal-directed learning and in building action-outcome associations. The participants demonstrated significantly stronger activation in two clusters in this region (Brodmann areas 25 and 10) when selecting actions in response to standard (vs. incongruent) cues. Both human and rodent studies provided evidence on the role of vmPFC (prelimbic cortex in rats) in goal-directed learning. For example, *in vivo* recordings from mice orbitofrontal cortex showed different activity patterns depending on whether lever presses were goal-directed or habitual^12^. In the same study, optogenetic manipulation of this region modified goal-directed responding in mice. Similarly, a number of imaging studies in humans demonstrated that vmPFC is implicated in encoding the expected value of a given action^24, 25^. Interestingly, specific roles have been previously suggested for anterior and posterior medial prefrontal cortex in ‘valuation’ and ‘conversion of value to choice’ respectively^15^. The two clusters we identified in our study might specifically be involved in these two distinct processes of the action-outcome system. Our results also identified another region implicated in goal-directed learning. PCC demonstrated stronger activation during standard cues. A previous study suggested that this region encodes the value of past decisions to guide future actions^26^. It was also suggested that PCC tracks the subjective value of alternative choices^27^. Overall, similar to de Wit *et al*. that also used a version of this task^18^, we identified vmPFC; however, we additionally found that two other regions, namely dmPFC and PCC, are also involved in goal-directed acquisition of stimuli. Taken together, our findings point to a value-driven goal-directed learning during standard trials and identify cortical regions associated with this distinct type of learning.

In contrast with goal-directed learning, habit learning is driven by stimulus-response associations^5^. As revealed by both post-scan tests, subjects learned to select the correct action in a way that is insensitive to the outcome value during incongruent trials. However, subjects demonstrated robust striatal activity during acquisition of both standard and incongruent pairs (Fig. S1). Numerous studies have reported dorsal striatal activation in acquisition and consolidation of instrumental behavior^28, 29^. In addition, early vs. late maps (Fig. 4) also suggest hemispheric specificity with right caudate predominantly active during acquisition of goal-directed actions, and left caudate mostly active during acquisition/consolidation of habitual actions. Thus, our finding might implicate a common neural substrate for early acquisition of instrumental behavior as well as a neural dissociation between goal-directed vs. habit acquisition within the striatum. In addition to heightened caudate activity, we also found enhanced bilateral anterior insula activation during incongruent trials. As part of the salience network, this region is sensitive to salient stimuli and is thought to facilitate access to the motor network^30^. Therefore, its enhanced activation during incongruent trials suggests that the insula is involved in detecting the saliency of incongruent stimuli with respect to standard ones.

In our imaging analyses, comparison of incongruent vs. standard cues did not reveal activation in brain areas typically associated with habit learning such as posterior putamen^16, 31^. However, we observed stronger bilateral activation in dorsal caudate during incongruent cues (vs. standard). It is important to note that even though the incongruent condition is thought to be mainly associated with stimulus-response learning^3^, stimulus-response associations are concurrently formed in the standard condition as well. Therefore, it is possible that regions revealed by the contrast ‘Incongruent vs. Standard’ underlie task-specific properties (such as incongruency) and not stimulus-response associations per se. A recent study implicated caudate in response to stimuli associated with elevated incongruency during a Stroop test^32^. Hence, the increased bilateral caudate activation may arise from the response conflict due to incongruency. Subjects’ greater responses to devalued outcomes and fewer responses to valuable outcomes during incongruent (vs. standard) trials in the Slip Test also support this possibility. Alternatively, the elevated caudate activation during incongruent trials may also result from the subjects’ attempt to resolve the response conflict generated by competing contingencies. Considering the lack of activation in striatal areas associated with habit learning, our findings may thus suggest that the incongruent condition may primarily be inducing conflict resolution rather than habitual acquisition of stimuli. Therefore, future work using human imaging might consider alternative approaches to task design to isolate habit learning.

In order to examine the temporal effects of learning, we contrasted early and late phases of learning for both discriminations. Our findings suggest that as actions become more automatic in the late phases, both dorsal (e.g., dmPFC) and ventral attention (e.g., insula) networks are less involved, which is consistent with previous results showing decreases in cortical dependency associated with practice^33^. Although it was observed exclusively during the standard condition, the enhanced activation in PCC in the late phase may also reflect reduced attention and involvement of the default mode network during this period. Our results do not support the idea of a shift in activity from ventral to dorsal striatum with increasing automaticity. Dorsal caudate showed stronger activation during the early stages of learning in both conditions. Increased activation in ventral putamen during outcomes in the late phase most likely reflects relatively higher points won due to rising accuracy in this part of the task. To the best of our knowledge, both insula/caudate activation during incongruent trials and activations during early vs. late phases of learning were not previously reported in studies using this discrimination learning task.

Approach/avoidance motivation trait is thought to affect subsequent action selection. For example, sensitivity to punishment (measured by BIS) influences a person’s tendency towards risky behavior^20^. The association we found between activation in the dmPFC during action selection at standard cues (compared to incongruent) and BIS scores may thus indicate inhibition of a particular key press during cues for which the outcome value is relevant. Interestingly, we also observed a significant correlation between the BIS scores and accuracy rates in standard trials, suggesting that inhibition is advantageous in goal-directed learning where outcome value is attended. Behavioral inhibition could potentially lead to more accurate choices by preventing the individual from making an impulsive choice. Indeed, higher error-related negativity (ERN) amplitude in EEG was previously reported in individuals with higher avoidance motivation^34^. ERN component is mainly associated with error monitoring and was demonstrated to predict academic performance among undergraduate students^35^. Therefore, better cognitive control and error monitoring could explain the correlation between behavioral inhibition and accuracy.

Our exploratory gender analysis found no gender differences in the traits measured by BIS/BAS and in learning accuracy, which is consistent with previous studies using large samples^36^. However, we found that females made relatively more outcome-sensitive responses during the Slip Test. We are not aware of any study that directly tested this. However, a previous study conducted in adolescents and young adults demonstrated that females make faster responses in go/no-go tasks using a stop signal^37^. Although our goal-directedness measure is more complicated than a simple go/no-go response and involves incorporating action-outcome associations, more efficient inhibitory response in females might be a contributing factor to this gender effect.

In conclusion, using a validated paradigm in fMRI we dissociate the brain regions that are involved in goal-directed and habit learning, and link behavioral inhibition to better performance during acquisition of goal-directed knowledge. Our findings highlight the central role of the medial prefrontal cortex in goal-directed learning, reveal the temporal dynamics of learning and emphasize the beneficial role of inhibition.

## Electronic supplementary material

Supplementary Information
