# Supplementary material for: Neural determinants of human goal-directed vs. habitual action control and their relation to trait motivation
Source: Sci Rep. 2017 Jul 20;7:6002. doi: 10.1038/s41598-017-06284-y (PMC5519538; doi:10.1038/s41598-017-06284-y)
Supplement: Supplementary file 1 — Supplementary Information [file 41598_2017_6284_MOESM1_ESM.pdf]

**Supplementary Information**

## Title

Neural determinants of human goal-directed vs. habitual action control and their relation to trait motivation

## Authors

Hamdi Eryilmaz[1]

Anais Rodriguez-Thompson[1]

Alexandra S. Tanner[1]

Madeline Giegold[1]

Franklin C. Huntington[1]

Joshua L. Roffman[1]

[1]Department of Psychiatry, Massachusetts General Hospital and Harvard Medical School, Boston, MA

## Corresponding author information

Name: Hamdi Eryilmaz

Address: Massachusetts General Hospital, 149 13th St, Room 2602-D, Charlestown, MA 02129, USA.

Email address: hamdi.eryilmaz@mgh.harvard.edu

Phone: +1 617 643 7462

Fax: +1 617 726 4078

**Supplementary Figures**

**Figure S1.** General effect of learning.

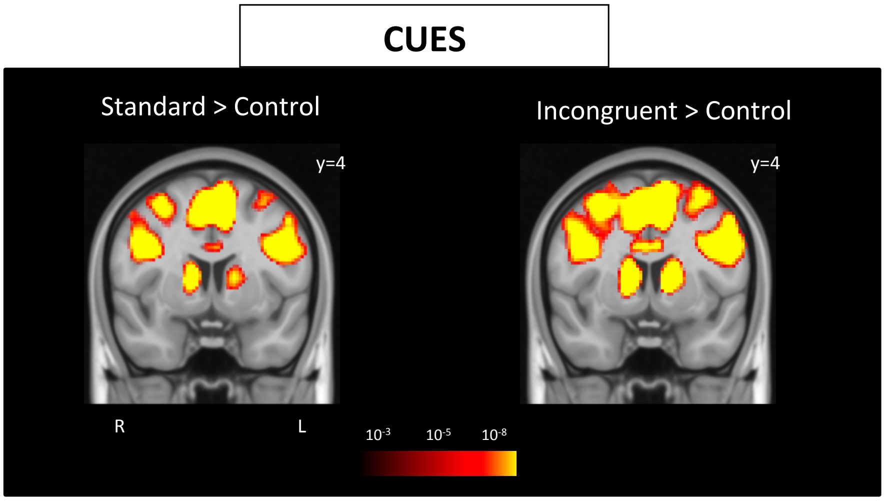


Subjects demonstrated strong activation in bilateral dorsal caudate and in the dorsal attention network (bilateral dorsolateral and dorsomedial prefrontal cortex, superior parietal lobule) in both learning conditions relative to the control condition.

**Figure S2.** Correlations among BIS/BAS-Drive, Goal-directedness, brain activation, and learning performance.

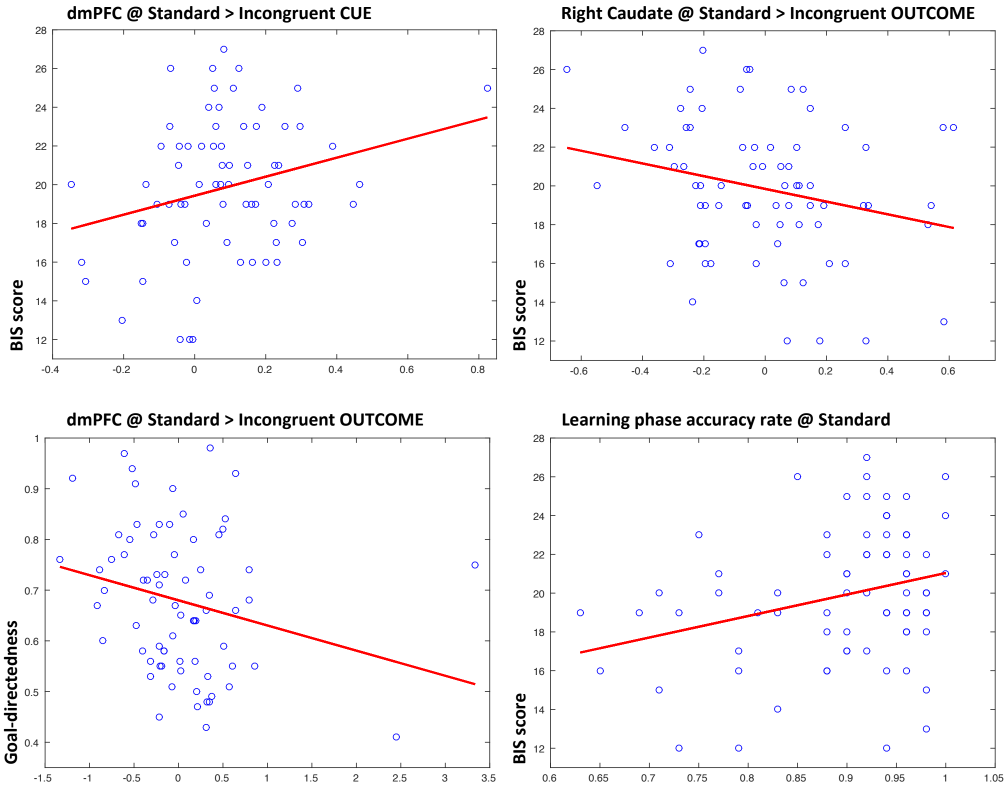


Correlations between behavioral scales and brain activation are depicted only for illustration purpose and they did not survive correction for multiple comparisons. BIS scores showed a nominally significant positive correlation with activation in dmPFC during standard cues and a negative correlation with activation in right caudate during standard outcomes. Goal-directedness measured in the Slip Test showed a nominally significant inverse correlation with dmPFC activation during standard outcomes. Finally, BIS scores significantly correlated with accuracy rates during acquisition of standard pairs.

## Supplementary Tables

**Table S1.** List of brain regions showing significant activation during early versus late phases of learning at standard and incongruent cues. Cluster size indicates the number of voxels surviving Monte Carlo corrected threshold. Coordinates and the p value are shown for the peak voxel in each region.

| Region (ROI) | Side | Coordinates | | | p-value | Cluster size (N) |
|---|---|---|---|---|---|---|
| | | x | y | z | | |
| ***Standard EARLY > Standard LATE*** | | | | | | |
| Caudate | L | -12 | -5 | 19 | $2.3x10^{-11}$ | 970 |
| Caudate | R | 11 | 7 | 13 | $5x10^{-9}$ | 1226 |
| Insula | R | 34 | 21 | -3 | $5.2x10^{-10}$ | 630 |
| dlPFC | R | 52 | 10 | 35 | $2x10^{-8}$ | 1988 |
| dmPFC | R | 2 | 35 | 41 | $3.4x10^{-8}$ | 855 |
| Insula | L | -30 | 19 | -3 | $4.9x10^{-8}$ | 422 |
| PrCG | L | -48 | 1 | 39 | $5.6x10^{-5}$ | 144 |
| ***Standard LATE > Standard EARLY*** | | | | | | |
| STG | R | 66 | -13 | -1 | $2.1x10^{-7}$ | 470 |
| PoCG | L | -14 | -31 | 79 | $5.5x10^{-7}$ | 405 |
| PCC | L | -4 | -43 | 23 | $10^{-6}$ | 1278 |
| PoCG | R | 16 | -31 | 77 | $1.3x10^{-6}$ | 251 |
| ***Incongruent EARLY > Incongruent LATE*** | | | | | | |
| dmPFC | R | 4 | 37 | 41 | $1.6x10^{-10}$ | 1376 |
| dlPFC | R | 46 | 15 | 51 | $2.1x10^{-8}$ | 708 |
| IFG | R | 56 | 11 | 18 | $3.1x10^{-8}$ | 800 |
| Caudate | R | 10 | -3 | 15 | $1.1x10^{-7}$ | 638 |
| Caudate | L | -8 | 5 | 9 | $1.7x10^{-7}$ | 514 |
| Insula | R | 34 | 21 | -11 | $1.8x10^{-6}$ | 336 |
| Insula | L | -32 | 25 | -5 | $10^{-5}$ | 368 |

*Abbreviations: dmPFC: dorsomedial prefrontal cortex, dlPFC: dorsolateral prefrontal cortex, IFG: inferior frontal gyrus, PCC: posterior cingulate cortex, PoCG: postcentral gyrus, PrCG: precentral gyrus, STG: superior temporal gyrus.*

**Table S2.** List of brain regions showing significant activation during early versus late phases of learning at standard and incongruent outcomes. Cluster size indicates the number of voxels surviving Monte Carlo corrected threshold. Coordinates and the p value are shown for the peak voxel in each region.

| Region (ROI) | Side | Coordinates | | | p-value | Cluster size (N) |
|---|---|---|---|---|---|---|
| | | x | y | z | | |
| ***Standard EARLY > Standard LATE*** | | | | | | |
| SMA | L | -2 | 3 | 61 | $8.1 \times 10^{-8}$ | 734 |
| dACC | | 0 | 13 | 28 | $1.6 \times 10^{-6}$ | 492 |
| dlPFC | R | 32 | 41 | 35 | $2.6 \times 10^{-5}$ | 323 |
| ***Standard LATE > Standard EARLY*** | | | | | | |
| Putamen | L | -16 | 5 | -9 | $7.9 \times 10^{-9}$ | 426 |
| Caudate | R | 12 | 9 | -3 | $3.7 \times 10^{-7}$ | 288 |
| MOcc | L | -28 | -89 | 17 | $1.4 \times 10^{-5}$ | 324 |
| ***Incongruent EARLY > Incongruent LATE*** | | | | | | |
| dlPFC | R | 34 | 41 | 29 | $1.5 \times 10^{-7}$ | 529 |
| dACC | L | -8 | 21 | 37 | $6.2 \times 10^{-6}$ | 758 |
| PrCG | L | -48 | 3 | 39 | $3.2 \times 10^{-5}$ | 398 |
| ***Incongruent LATE > Incongruent EARLY*** | | | | | | |
| Putamen | L | -20 | 5 | -7 | $4.2 \times 10^{-7}$ | 388 |

*Abbreviations: dACC: dorsal anterior cingulate cortex, dlPFC: dorsolateral prefrontal cortex, MOcc: middle occipital cortex, PrCG: precentral gyrus, SMA: supplementary motor area.*

**Supplementary Results**

**<u>GLM results.</u>** In order verify that the conditions *standard* and *incongruent* induced the predicted learning effects, we contrasted these two conditions with the activation during the control condition, which consisted of the visual and motor properties of the learning conditions.

*General learning effects.* The contrast 'Standard Cue vs. Control Cue' showed strong activation in distributed brain areas including the dorsal attention network (bilateral dlPFC, dmPFC, superior parietal lobule), bilateral insula, bilateral dorsal caudate and thalamus and lateral occipital cortex (Fig. S1). Widespread cortical activation suggests that learning conditions required more attentional resources than the control condition, whereas substantial striatum activation hints at learning effects. 'Incongruent Cue vs. Control Cue' activated similar brain areas as above with slightly larger clusters in the striatum and in the dorsal attention network.
